# Protein Variability Patterns in Ovarian Serous Carcinoma

**DOI:** 10.3390/ijms27188012

**Published:** 2026-09-09

**Authors:** Arina I. Gordeeva, Anna A. Kliuchnikova, Anna S. Kozlova, Svetlana N. Tarbeeva, Elena S. Zorina, Elizaveta V. Sarygina, Elena A. Glagoleva, Elena A. Ponomarenko, Ekaterina V. Ilgisonis

**Affiliations:** 1 Institute of Biomedical Chemistry, 119121 Moscow, Russia; arina.atom@gmail.com (A.I.G.); el.zorina@ibmc.msk.ru (E.S.Z.); lizalesa@gmail.com (E.V.S.); 2463731@gmail.com (E.A.P.); 2 Sirius University of Science and Technology, 1 Olympic Ave., 354340 Sochi, Russia; 3 Sechenov Centre for Motherhood and Childhood, Clinical Centre, I.M. Sechenov First Moscow State Medical University (Sechenov University), 119435 Moscow, Russia; glagoleva.md@mail.ru

**Keywords:** proteomics, coefficient of variation, protein variability, ovarian cancer, HGSC, label-free proteomics, mass spectrometry, tumor heterogeneity

## Abstract

Protein variability patterns in cancer reflects both technical variation and biological heterogeneity and may provide information beyond mean abundance changes. We analyzed protein-level coefficients of variation across four ovarian cancer proteomic datasets, comparing controls and cancer samples. We analyzed protein-level coefficients of variation (CVs) across four ovarian cancer proteomic datasets, comparing control and tumor samples. Among 7476 proteins, the median CV increased from 93% in controls to 126% in cancer, and the distributions differed significantly between the two groups (Wilcoxon *p* < 2.2 × 10^−16^; Kolmogorov–Smirnov *p* = 4.2 × 10^−242^). The largest increases in variability were observed among proteins with low inter-individual variability in controls, whereas proteins that were already highly variable showed more heterogeneous behavior, including decreases in CV. Thus, cancer was associated not only with an overall increase in variability but also with a redistribution of proteins across variability states. Gene Ontology analysis revealed functional differences between stable and highly variable proteins. Stable proteins were predominantly associated with intracellular, organelle-related, biosynthetic, and metabolic processes, whereas highly variable proteins were more frequently linked to membrane, vesicle-related, and signaling functions. Proteins that remain stable from the control to cancer state, as well as those that lose this stability during tumor development, may therefore be of particular interest. These results support protein variability as an additional analytical dimension alongside fold-change analysis for describing proteome instability and tumor heterogeneity. Inter-individual variation in protein abundance may reflect biological heterogeneity that is not captured by conventional comparisons of mean expression levels. Variability analysis complements conventional abundance-based approaches and provides an additional framework for characterizing tumor proteome heterogeneity.

## 1. Introduction

Mass spectrometry-based proteomics has become a central technology for studying cancer biology, enabling biomarker discovery and systems-level investigation of molecular regulation [1,2]. Large-scale proteomic datasets now allow quantitative measurement of thousands of proteins across clinical samples, providing insight into tumor-associated pathways, cellular states, and disease mechanisms. The accessibility of existing data, exemplified by PRIDE—the premier global repository for mass spectrometry-based proteomics data [3]—significantly enhances the ease of reusing, analyzing, and sharing these valuable resources.

Most proteomic studies focus on differential abundance analysis, comparing proteins between conditions based on their average expression levels [4]. Candidate markers are typically selected according to fold change, statistical significance, and functional relevance [5]. While this approach is powerful, it captures only one dimension of the data: differences in central tendency between groups.

Protein abundance, however, is not defined solely by its mean value. Individual proteins also vary considerably between patients within the same biological condition [6]. This variability is often treated as unwanted noise arising from technical variation, sample handling, missing values, or uncontrolled biological heterogeneity. However, rather than being mere analytical artifacts, shifts in variability may reflect biological heterogeneity in addition to technical and pre-analytical sources of variation. For instance, the concept of a ‘core proteome’, defined as comprising proteins with minimal interindividual coefficient of variation (CV) in healthy populations, has been established as a critical baseline for biomarker discovery [7]. Proteins exhibiting such tightly regulated baseline expression are particularly promising as diagnostic targets, because a low physiological variance inherently maximizes the statistical power to detect significant pathological deviations. Standard workflows therefore aim to minimize variability through normalization, filtering, transformation, and batch correction [8,9].

In cancer, however, inter-individual protein variability may carry biological meaning [10,11]. Tumors are intrinsically heterogeneous systems composed of malignant cells, stromal components, immune infiltrates, extracellular matrix elements, and variable vascular and metabolic microenvironments [12]. Importantly, this heterogeneity is reflected not only at the molecular level but also in clinically relevant characteristics, including differential response to chemotherapy and targeted therapies among patients with histologically similar tumors. Cancer-associated proteome remodeling may therefore manifest not only as shifts in mean protein abundance but also as altered protein-level variability across samples. Characterizing variability patterns may thus represent an important step toward identifying proteomic signatures associated with clinically distinct tumor subgroups. We selected high-grade serous ovarian cancer (HGSC) as a model system because it is characterized by marked molecular and clinical heterogeneity, high mortality, late-stage diagnosis, and the absence of robust single-protein biomarkers [5]. This heterogeneity also extends to the microenvironment, with distinct transcriptional and proteomic programs reported between ascitic and solid tumor compartments [13].

The coefficient of variation (CV) provides a normalized measure of dispersion,

CV = (σ/μ) × 100%,

where σ is the standard deviation and μ is the mean abundance. CV enables comparison of relative variability across proteins with different abundance ranges. In proteomics, CV is routinely used to assess reproducibility or quantify technical variation [14,15]. When calculated across biological samples, however, CV can also capture inter-individual heterogeneity and disease-associated instability [6]. In a study conducted by Geyer et al. [15], it was shown that plasma proteomics achieved a technical coefficient of variation (CV) of less than 20%, indicating that biological variation is the predominant source of variability. Furthermore, a meta-analysis of targeted quantitative plasma screens produced a reference kit for health analytics. In that meta-analysis, 53 of the 296 quantified plasma proteins met the criteria for a stable healthy plasma proteome, being detected in at least 70% of samples with an inter-individual coefficient of variation below 40% [6].

The analysis of variability as an independent biological feature was previously developed in transcriptomics. Differential variability analysis methods account for the fact that observed dispersion depends on the mean signal level and includes both biological and technical components. This dependence can be particularly pronounced for low-intensity features, for which relative variability is often artificially inflated. Consequently, modern transcriptomic approaches combine normalization, mean–variance trend modeling, and estimation of residual variability [16,17,18]. A similar principle applies to proteomic data, where the coefficient of variation should be interpreted in light of mean protein intensity, detection frequency, and the technical characteristics of the measurement [19,20].

In the present study, we introduce the analysis of protein variability patterns as a distinct proteomic feature. We therefore evaluated CV as a biological dispersion measure alongside conventional protein abundance, rather than as a quality-control metric alone. We performed a pooled analysis of four publicly available ovarian cancer proteomic datasets spanning tissue and secretory proteome sources [21,22,23,24] and calculated protein-level CV separately for the control and cancer groups. Differential variability was summarized using signed ΔCV and log2(CV_Cancer_/CV_Control_), with project-stratified permutation testing, bootstrap confidence intervals, split-half reproducibility analysis, and empirical-Bayes shrinkage. Fixed CV groups (G1–G5) were used to describe transitions and define pre-specified subsets; protein-level conditional permutation testing was performed across all 4024 proteins classified as stable in control. To evaluate complementarity, project-adjusted differential-abundance analysis was performed for 4695 proteins detected in at least 50% of both groups; the results and CV annotations are provided in Supplementary Figure S1 and Supplementary Table S1.

The aim of this work was therefore not to establish increased proteomic variability as a cancer-specific phenomenon, but to determine whether changes in protein-level CV are systematically organized in ovarian cancer and whether they provide information complementary to conventional abundance-focused analysis.

## 2. Results

### 2.1. Dataset Overview and CV Coverage

A total of 7476 proteins satisfied the primary detection criterion (≥3 non-zero observations in both the control and cancer groups, without imputation) and had valid CV estimates in both conditions. Sensitivity analyses using stricter detection-proportion thresholds (50%, 70% and 80% in both groups; *n* = 4695, 3252 and 2353 proteins, respectively) yielded consistent results (Supplementary Figures S2 and S3 and Supplementary Table S2).

The dataset comprised 180 biological sample-level profiles, including 44 control and 136 cancer samples, providing sufficient statistical depth for stable estimation of coefficient of variation (CV) distributions and variability-based analyses.

**Figure 1:**
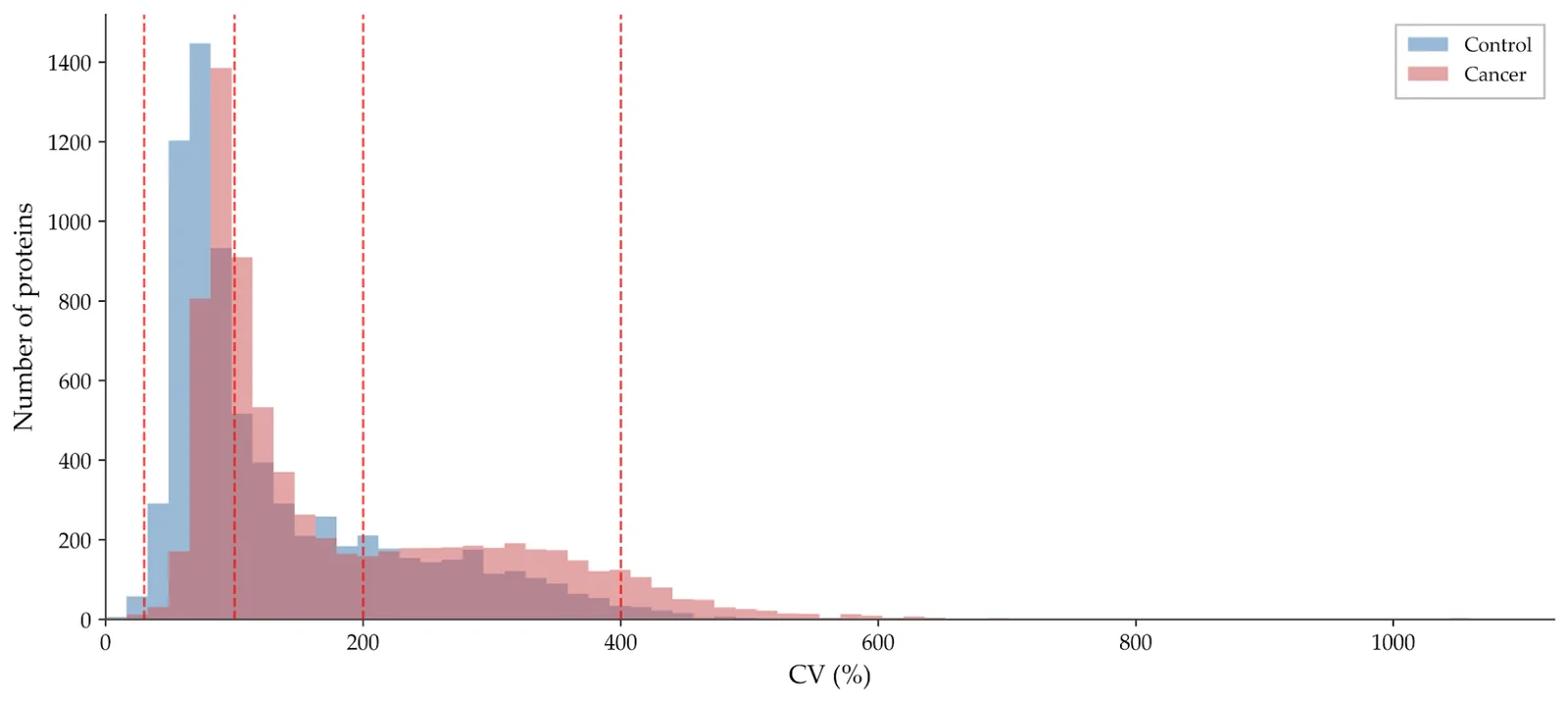
Distribution of protein coefficients of variation (CVs) in control and cancer samples. Histograms show protein-level CVs calculated from normalized, non-log-transformed intensities for 7476 proteins quantified in both conditions (44 control and 136 cancer sample-level profiles). Vertical dashed lines indicate predefined CV-group thresholds (G1–G5), shown for visualization only. Median CVs were 93% in controls and 126% in cancer samples; the median paired signed difference was +27.6 percentage points. Wilcoxon signed-rank test, *p* < 2.2 × 10^−16^ (*n* = 7476); Kolmogorov–Smirnov test, D = 0.271, *p* = 4.2 × 10^−242^. Vertical dashed lines indicate the predefined CV thresholds used to classify proteins into five variability groups: G1, 0–30% (very low variability); G2, 30–100% (low variability); G3, 100–200% (intermediate variability); G4, 200–400% (high variability); and G5, >400% (very high variability).

As shown in Figure 1, the distribution of protein-level CV values differs substantially between control and cancer samples (median CV: 93% in control vs. 126% in cancer; median ΔCV = +27.6 percentage points; Wilcoxon signed-rank test, *p* < 2.2 × 10^−16^; Kolmogorov–Smirnov D = 0.271, *p* = 4.2 × 10^−242^). The cancer group distribution is shifted toward higher CV values and exhibits increased dispersion relative to the control group. In addition, cancer samples display a pronounced expansion of the right tail, with a larger proportion of proteins occupying high-variability ranges (CV > 200% and CV > 400%), reflecting a global increase in protein-level variability and broader dispersion across samples. In contrast, control samples are primarily concentrated within low and intermediate variability ranges. This shift affects the overall distribution rather than being driven by a small number of extreme outliers.

To assess project-specific behavior, CV distributions were summarized separately for each dataset. Median CV was higher in cancer for PXD009655 (control, 174.95%; cancer, 339.05%; median ratio, 1.94; bootstrap 95% CI, 1.22–5.12) and PXD033741 (70.31% versus 88.74%; ratio, 1.26; 95% CI, 1.20–1.43). The point estimate was also higher in PXD025864 (48.52% versus 68.97%; ratio, 1.42), but its confidence interval was wide and crossed 1 (95% CI, 0.71–2.61). PXD012998 showed no directional increase (56.61% versus 55.42%; ratio, 0.98; 95% CI, 0.80–2.12). Supplementary Figures S4 and S5 show the project-specific control–cancer CV relationships and summary values; Supplementary Table S3 provides sample sizes, protein counts, medians, ratios, and bootstrap confidence intervals. Because several projects contain very few control samples, these comparisons are treated as descriptive sensitivity analyses rather than independent validation.

Leave-one-project-out (LOPO) analysis then recomputed the valid protein universe, CV values, and G1–G5 assignments after excluding each project. Median cancer CV remained above median control CV in all four reduced datasets. After Benjamini–Hochberg correction across the eight pre-specified primary tests, the complete CV-distribution endpoint remained significant only after excluding PXD012998 (q = 0.0024) or PXD025864 (q = 0.0424), but not after excluding PXD009655 or PXD033741 (q = 0.8283 for both). Redistribution of the marginal G1–G5 composition was not significant after any exclusion (all q ≥ 0.481). Secondary Stable → Stable and Stable → Variable endpoints were also not significant after correction (all FDR ≥ 0.234). Thus, LOPO indicates substantial project heterogeneity and sensitivity to study composition, not uniform replication across datasets (Supplementary Figure S6 and Supplementary Table S4).

### 2.2. Distribution of CV Within Baseline-Defined Groups

**Figure 2:**
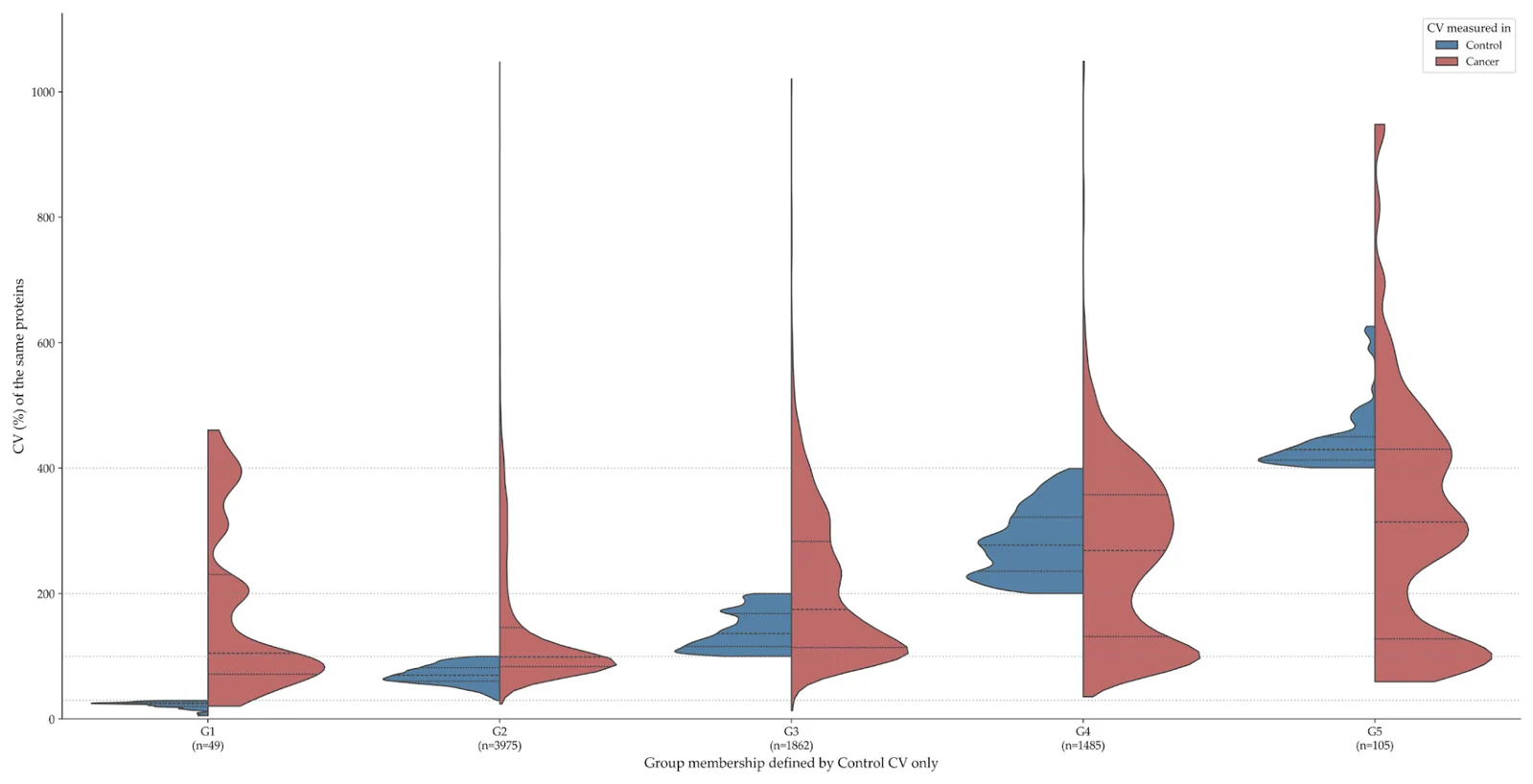
Distribution of protein-level CVs across control-defined groups. Group membership was defined by the control CV; blue violins show control CVs and red violins show cancer CVs for the same proteins. Paired Wilcoxon signed-rank tests (two-sided raw *p* values; all five comparisons remained significant after Benjamini–Hochberg correction): G1, *n* = 49, *p* = 1.07 × 10^−14^; G2, *n* = 3975, *p* < 1 × 10^−300^; G3, *n* = 1862, *p* = 2.19 × 10^−102^; G4, *n* = 1485, *p* = 7.05 × 10^−8^; and G5, *n* = 105, *p* = 5.53 × 10^−10^. Horizontal dotted lines indicate the predefined CV-group boundaries (G1–G5: <30%, 30–100%, 100–200%, 200–400%, and >400%), shown for visualization only.

To assess how variability changes within baseline-defined stability groups, CV distributions were examined using violin plots stratified by control-defined groups (G1–G5) (Figure 2). Violin plots were used to preserve the full shape of the underlying distribution through kernel density estimation, allowing visualization of features such as multimodality, asymmetry, and local modes that may be obscured by summary statistics.

Each violin represents the full distribution of CV values, including density, interquartile range, and extreme values. Control-group distributions follow the expected stepwise pattern based on grouping thresholds. Cancer-group distributions are broader, with pronounced expansion in low-variability groups (G1–G2) and partial compression in the highest-variability group (G5). These patterns indicate a baseline-dependent redistribution of protein variability in the cancer group.

By construction, control-group distributions show clear stepwise separation across groups, reflecting threshold-based classification. In contrast, cancer-group distributions are broader and less constrained within each group.

In low-variability groups (G1–G2), cancer-group samples demonstrate a strong increase in dispersion. Median CV increases from 24.5% to 104.9% in G1 and from 69.9% to 98.9% in G2. This is accompanied by a marked widening of interquartile ranges and the appearance of extended upper tails, with upper tails extending to approximately 400–1000%. Project-stratified null simulation and empirical-Bayes shrinkage were therefore used to assess whether this pattern could be explained by label structure or regression to the mean (Statistical Support for the Main Transition Classes; Supplementary Table S5). Proteins from the G1 group showing a pronounced CV increase in cancer may warrant further evaluation as indicators of proteomic instability, although candidate-level interpretation requires independent validation.

Intermediate groups (G3–G4) show similar but less pronounced changes. Median CV increases in G3 (136.1% to 174.7%), while in G4 the median remains comparable (277.2% to 268.5%), but the distribution becomes broader and more asymmetric, indicating increased heterogeneity without a strong shift in central values.

In contrast, the highest-variability group (G5) shows partial compression in cancer. The median CV decreases from 429.5% to 314.0%, although the distribution remains wide (Q1 = 127.6%, Q3 = 430.1%, maximum ≈ 950%), indicating persistent variability despite reduced central tendency. Notably, G5 includes proteins with well-established roles in reproductive physiology, such as MUC1, S100P, and LTF, whose expression is tightly regulated by circulating estradiol and progesterone throughout the menstrual cycle [25,26,27]. In healthy premenopausal controls, the CVs for these proteins may be influenced by the cycle phase at the time of sampling. In ovarian cancer, disruption of systemic endocrine homeostasis or autonomous, hormone-independent production of these proteins by the tumor may lead to pronounced shifts in variability.

Importantly, these changes affect the full distribution rather than only median values, including expansion of spread, elongation of tails, and redistribution of density within groups.

Overall, these results indicate that cancer is associated with a redistribution of variability across baseline-defined groups. Low-variability proteins tend to become more variable, whereas highly variable proteins tend to become less variable. The extreme low-to-high class changed from 733 proteins in the raw analysis to 751 after empirical-Bayes shrinkage, whereas the extreme high-to-low class decreased from 219 to 123 proteins; the two directions should therefore not be interpreted as equally robust evidence of biological reorganization (Statistical Support for the Main Transition Classes; Supplementary Table S5).

### 2.3. Transitions Between Variability States

**Figure 3:**
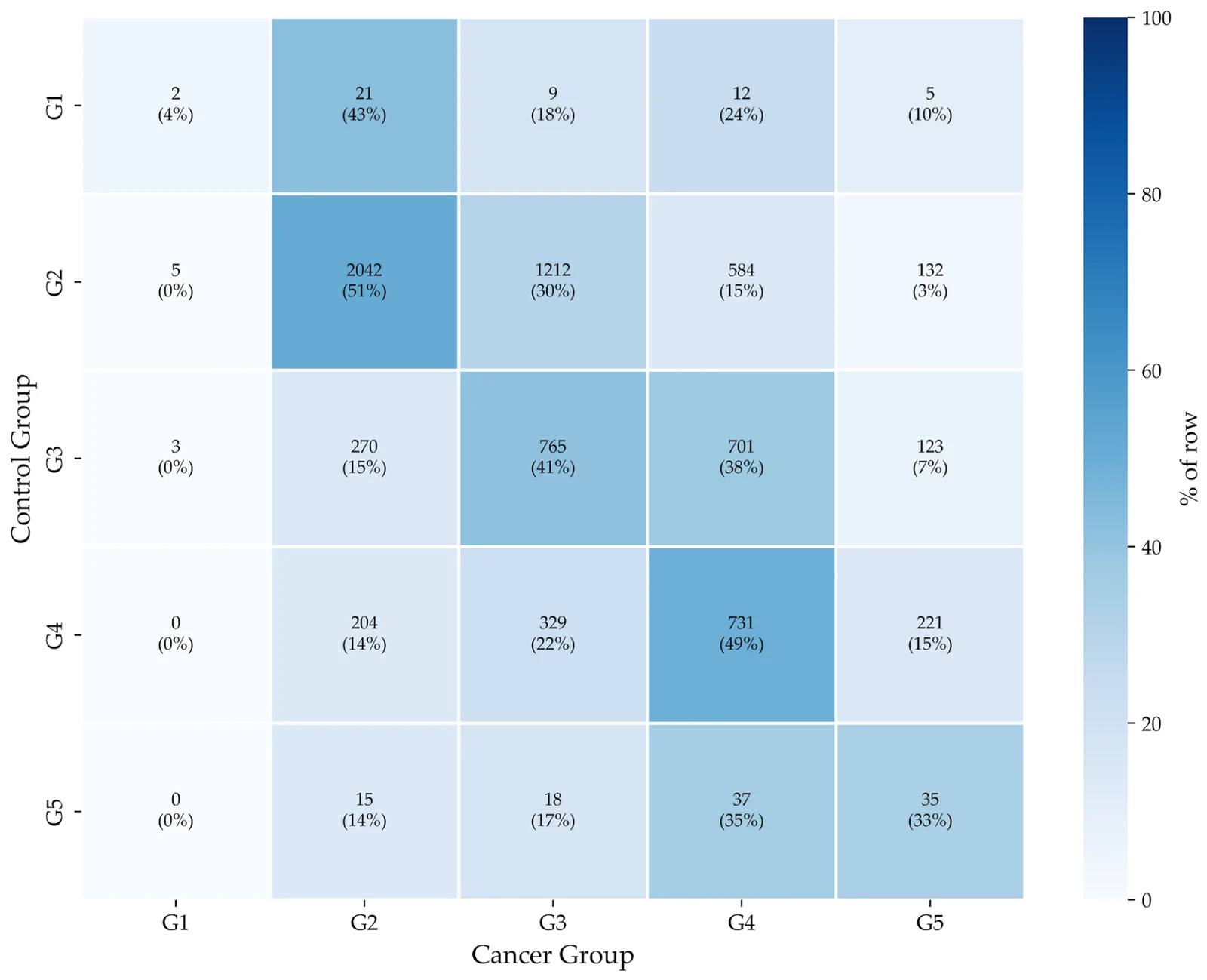
Transition matrix of CV-defined protein groups (control to cancer). Heatmap showing redistribution of proteins between variability groups (G1–G5) from control (rows) to cancer (columns). Values represent the number of proteins and the percentage within each control-defined group. The matrix highlights upward transitions in low-variability groups and downward transitions in high-variability groups; bootstrap confidence intervals for transition-class proportions are provided in Supplementary Figure S7 and Supplementary Table S5.

To examine how protein variability changes between conditions, transitions between control- and cancer-defined CV groups were analyzed using a transition matrix (Figure 3).

In the lowest-variability group (G1), only 4.1% of proteins remain in the same group, while most shift toward higher variability: 42.9% move to G2, 18.4% to G3, 24.5% to G4, and 10.2% to G5. This indicates that many proteins with low variability in the control group become more variable in the cancer group.

In G2, 51.4% of proteins remain in the same group, while 30.5% shift to G3, 14.7% to G4, and 3.3% to G5. Transitions toward lower variability are minimal (0.1%), indicating a net increase in variability within this group.

Intermediate group G3 shows bidirectional changes. While 41.1% of proteins remain in G3, 44.2% shift to higher-variability groups (37.6% to G4 and 6.6% to G5), and 14.5% move to G2. This reflects heterogeneous behavior within this group.

In G4, 49.2% of proteins remain in the same group, while 22.2% shift to G3 and 13.7% to G2. At the same time, 14.9% move to G5, indicating that both increases and decreases in variability occur.

In the highest-variability group (G5), only 33.3% of proteins remain in G5. The majority shift toward lower variability: 35.2% move to G4, 17.1% to G3, and 14.3% to G2. No transitions to G1 are observed.

Overall, the observed transition matrix descriptively showed that proteins with low variability in control often moved toward higher-CV groups, whereas proteins with high variability often moved toward lower-CV groups. However, this pattern did not by itself establish systematic biological reorganization, because the pre-specified primary transition proportions did not exceed the project-stratified null expectation after FDR correction. The transition counts are therefore interpreted descriptively and require validation in independent, clinically annotated cohorts.

#### Statistical Support for the Main Transition Classes

Project-stratified label permutations tested the two pre-specified primary conditional transition proportions. After Benjamini–Hochberg correction, neither the Stable Retained (2070 proteins; FDR = 0.422) nor Destabilized (733 proteins; FDR = 0.071) classes exceeded the null expectation. Empirical-Bayes shrinkage was applied to all 7476 proteins before reassignment of CV groups: Stable Retained changed from 2070 to 2014, Destabilized from 733 to 751, and Stabilized from 219 to 123. Thus, the Destabilized class was robust to CV shrinkage but was not statistically enriched relative to the primary null. Detailed results and a consolidated statistical summary are provided in Supplementary Table S5.

Separately, protein-level conditional differential-variability screening was applied to all 4024 proteins classified as Stable in control. Of the 733 proteins descriptively classified as Destabilized, 566 met the adequacy threshold (at least 1000 accepted conditional permutations) and 95 had Benjamini–Hochberg q < 0.05 across the complete testing family. These 95 proteins are reported as exploratory statistically prioritized candidates requiring independent validation, not as validated biomarkers; complete effect sizes and permutation results are provided in Supplementary Table S6.

### 2.4. Protein-Level Patterns in Extreme Variability Groups

**Figure 4:**
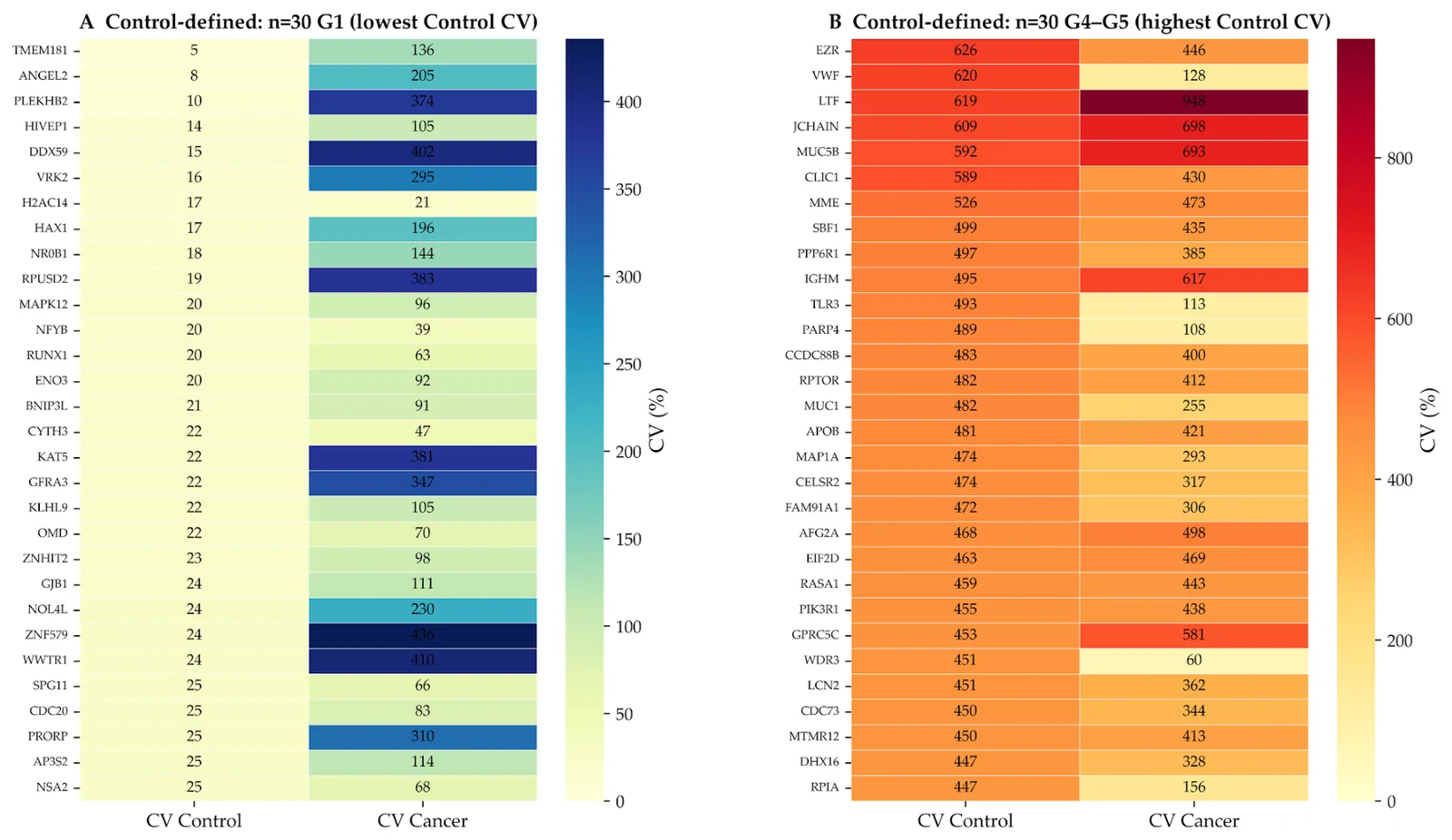
Extreme CV proteins with gene-symbol labels (control-defined panels). Heatmaps of CV (%) in control and cancer for the 30 lowest-control-CV G1 proteins (**A**) and the 30 highest-control-CV G4–G5 proteins (**B**), ranked within the common set of proteins with valid CV in both conditions (*n* = 7476). Row labels show gene symbols; UniProt accessions are used only when no symbol is available or when symbols are duplicated. Cell values are observed CV (%); color scales differ between panels because G1 and G4–G5 occupy different CV ranges. Cancer-defined extreme rankings are provided in Supplementary Table S7.

To examine protein-level variability patterns, heatmaps were constructed for proteins from the extreme control-defined CV groups (Figure 4).

In the stable subset (A), proteins exhibit consistently low CV values in the control group, reflecting tight regulation across samples. In the cancer group, many of these proteins show a marked increase in variability, often accompanied by broader dispersion across the dataset. This pattern is consistent with the redistribution reported in Section 2.2 and remained evident after shrinkage, although individual protein assignments require cautious interpretation because split-half stability was moderate (Supplementary Tables S5 and S8).

In contrast, the highly variable subset (B) shows a different pattern. These proteins display very high CV values in the control group, including examples such as EZR (626.1%), VWF (619.7%), LTF (618.6%), JCHAIN (608.5%), and MUC5B (591.7%). In the cancer group, variability remains high overall, but the response is not uniform. Some proteins retain or further increase their variability (e.g., LTF: 948.1%, JCHAIN: 698.4%, MUC5B: 692.7%), while others show a marked reduction compared to control (e.g., VWF: 127.6%, TLR3: 112.6%, PARP4: 107.6%).

Ranking the same protein set by cancer-defined CV instead (panels C–D) reveals a marked asymmetry. Only 10 proteins met the G1 threshold (CV < 30%) under the cancer-defined classification, compared with the full complement of 30 proteins obtainable under the control-defined ranking (panel A). This asymmetry indicates that very few proteins are simultaneously among the most stable when variability is assessed within the cancer group itself, consistent with the broader, less constrained cancer-group CV distributions described in Section 2.2. Notably, several of these cancer-defined stable proteins had comparatively high control CV (e.g., H2BC14: 173% in control vs. 13% in cancer; EBI3: 146% vs. 22%), indicating a pronounced reduction in variability for this specific subset in cancer despite substantial baseline heterogeneity.

The cancer-defined highly variable subset (D) substantially overlaps with, but is not identical to, the control-defined highly variable subset (B): while proteins such as LTF, JCHAIN, and MUC5B appear in both rankings, panel D additionally includes proteins with comparatively low or moderate control CV that show a pronounced increase in cancer (e.g., SDCBP: 74% → 1047%; CHMP2A: 50% → 925%; SENP3: 41% → 641%), consistent with the destabilization pattern described for the G1 group in Section 2.2.

Importantly, proteins within the same variability group do not behave identically. Both increases and decreases in CV are observed, indicating that variability changes occur at the level of individual proteins rather than uniformly across groups, and that control-defined and cancer-defined rankings capture partially distinct, complementary aspects of this heterogeneity.

### 2.5. Functional Composition Across Variability Groups

**Figure 5:**
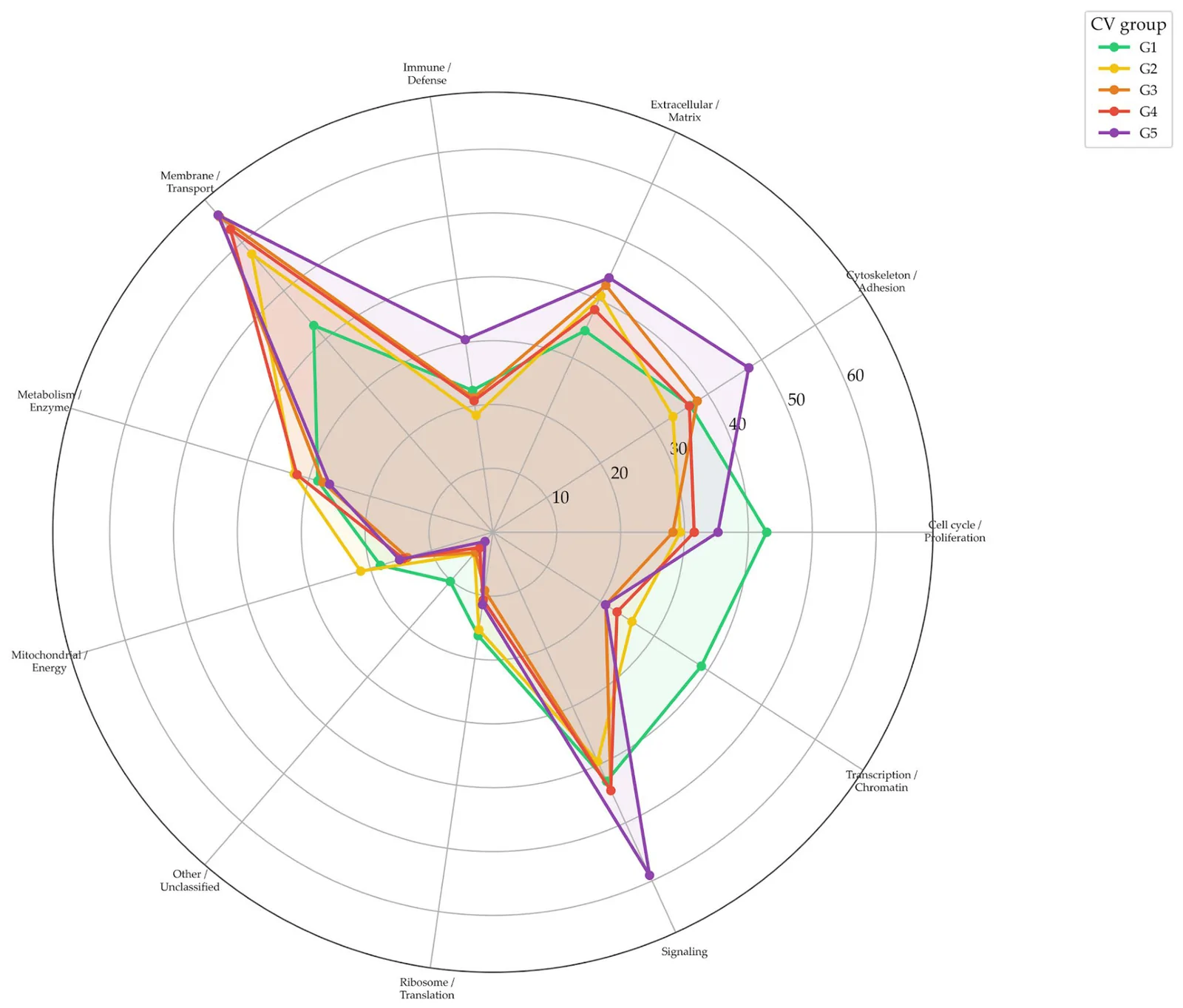
Functional composition across control-defined CV groups. Radar plot showing the percentage of proteins assigned to functional categories across CV groups (G1–G5), defined by control CV values. Each point represents the proportion of proteins within a category in a given group. Category assignment is multi-label; percentages within a group do not sum to 100%. Source values are provided in Supplementary Table S9.

To assess whether protein variability is associated with functional organization, the distribution of functional categories was examined across control-defined CV groups (G1–G5) (Figure 5). The figure demonstrates non-uniform changes in functional composition across variability levels, including enrichment of extracellular, membrane, and signaling proteins in higher-variability groups and reduced contribution of transcription/chromatin and ribosome-related proteins.

Proteins were grouped according to their control CV values, and for each group the proportion of proteins assigned to functional categories was calculated. The results show that functional composition changes across variability groups, though not always monotonically.

Extracellular/matrix proteins show an overall increase toward higher variability groups, rising from 34.7% in G1 to 43.8% in G5, with intermediate values of 40.7% (G2), 42.5% (G3), and a slight decrease to 38.3% in G4. This indicates a general, though non-monotonic, enrichment of extracellular proteins in higher-variability groups.

In contrast, transcription/chromatin proteins show a clear decrease from low- to high-variability groups, declining from 38.8% in G1 to 21.0% in G5. Intermediate values remain consistently lower than G1 (25.9% in G2, 21.0% in G3, 23.1% in G4), indicating reduced representation in more variable groups.

Membrane/transport proteins represent the largest category across most groups and increase substantially from 42.9% in G1 to 65.7% in G5, with high intermediate values (57.7% in G2, 65.4% in G3, 62.8% in G4). This suggests strong enrichment of membrane-associated proteins in higher-variability groups.

Signaling proteins show a similar pattern, increasing from 42.9% in G1 to 59.0% in G5, with relatively stable intermediate values (39.5% in G2, 44.5% in G3, 44.4% in G4) before the marked rise in the most variable group.

Cytoskeleton/adhesion proteins remain relatively stable across G1–G4 (33.5–38.0%) but increase to 47.6% in G5, indicating higher representation in the most variable group.

Immune/defense proteins show moderate variation across intermediate groups (18.5–21.3%) but increase to 30.5% in G5 compared to 22.4% in G1, suggesting greater representation of immune-related proteins among highly variable proteins.

In contrast, ribosome/translation proteins are highest in G1 (16.3%) and G2 (15.4%), reach a minimum in G3 (9.2%), and remain lower than G1–G2 in G4–G5 (10.7% and 11.4%, respectively). Mitochondrial/energy proteins follow a related pattern, decreasing from 18.4% in G1 to 14.0–15.2% in G3–G5. For low-abundance proteins, these trends are likely attributable at least in part to technical rather than purely biological factors.

The transition from low to high variability (G1 to G5) is associated with functional redistribution rather than uniform changes across all protein classes. Higher-variability groups are relatively enriched in extracellular, membrane, signaling, cytoskeletal, and immune-related proteins, whereas lower-variability groups contain a higher proportion of transcription/chromatin and ribosome-related proteins.

### 2.6. Functional Organization of Variability Across GO-Defined Protein Subsets

**Figure 6:**
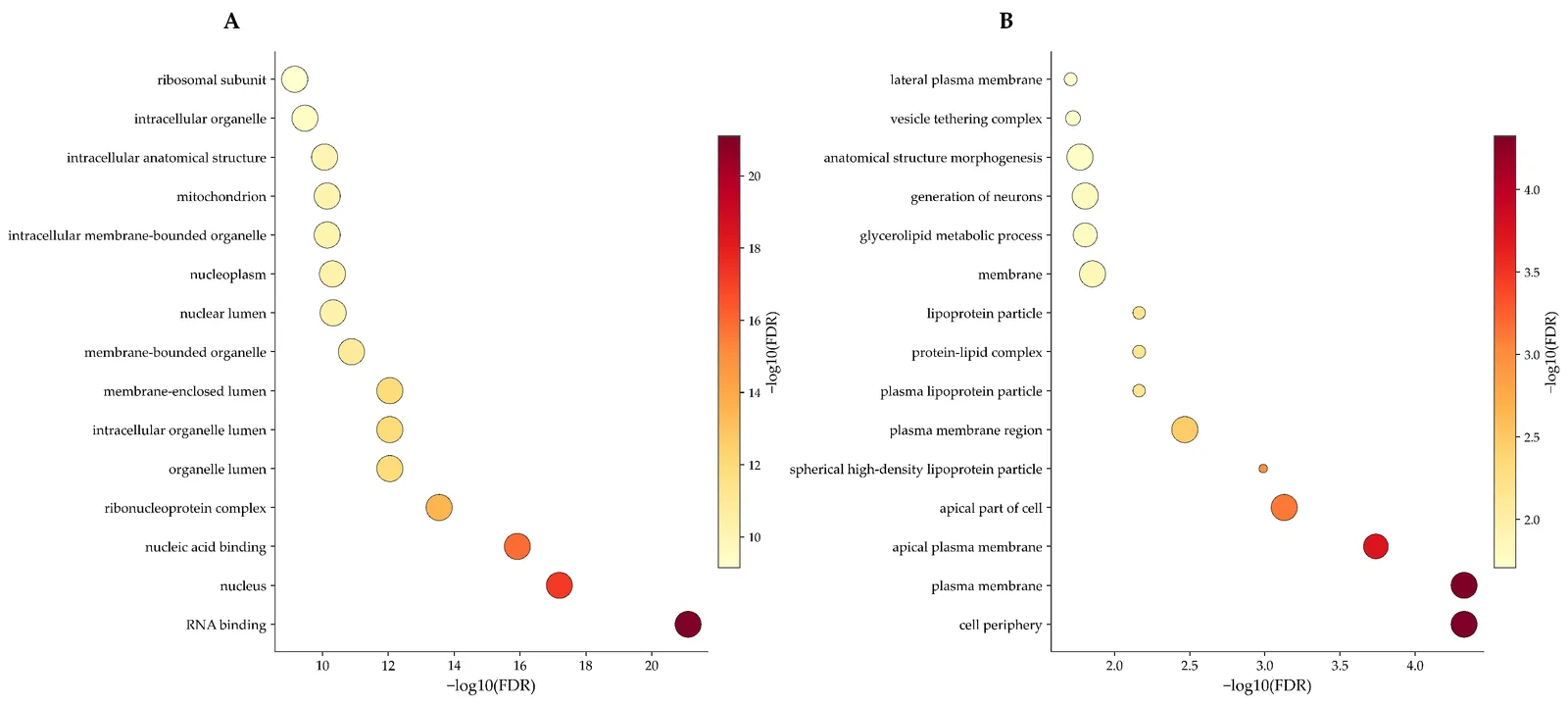
GO enrichment across variability-defined protein subsets. Dot plots show enriched GO terms for two CV-defined protein subsets: (**A**) Stable G1–G2 and (**B**) Variable G4–G5. The *x*-axis indicates enrichment significance (−log10(FDR)); larger values correspond to stronger enrichment. Dot size represents intersection size (number of proteins from the queried subset assigned to each GO term). Terms are ranked by significance and include GO:BP, GO:MF, and GO:CC categories.

To define the biological structure underlying CV-based variability states, GO enrichment analysis was performed for four protein subsets: Stable G1–G2, Variable G4–G5, Destabilized (G1 + G2 to G4 + G5), and Stabilized (G4 + G5 to G1 + G2). Significant GO enrichment (FDR < 0.05) was identified for the Stable G1–G2 and Variable G4–G5 subsets shown in Figure 6A,B. No GO term met this threshold for the Destabilized subset, whereas two terms were significant for the Stabilized subset: nucleic acid binding (FDR = 2.93 × 10^−4^; intersection size = 82) and rRNA metabolic process (FDR = 0.0322; intersection size = 18). Complete results for all four subsets are provided in Supplementary Table S10. Statistical significance is presented as −log10(FDR) on the *x*-axis.

In the Stable G1–G2 subset, the significant GO terms were mainly related to intracellular and organelle components, biosynthetic and metabolic processes, and nucleic acid-binding functions. This pattern indicates that proteins are predominantly associated with intracellular and organelle organization, as well as with biosynthetic and metabolic activities.

For Variable G4–G5, GO enrichment was shifted toward membrane- and cell-periphery-associated components, with additional terms related to lipid-associated structures, vesicle-related functions, and signaling.

Taken together, GO enrichment analysis revealed distinct functional profiles for the Stable G1–G2 and Variable G4–G5 subsets. No term passed FDR < 0.05 for the Destabilized subset. The Stabilized subset yielded two significant terms—nucleic acid binding and rRNA metabolic process—although this limited result should be interpreted cautiously because the query contained only 216 mapped gene symbols.

These results indicate that variability across the proteome is functionally structured, with the Stable and Variable subsets showing distinct and statistically robust enrichment profiles. Among the transition subsets, Destabilized proteins showed no significant GO terms, whereas Stabilized proteins showed two significant terms. Given the smaller transition-set sizes and the shrinkage sensitivity of the Stabilized classification, these transition-specific enrichment results require cautious interpretation and independent validation.

### 2.7. Functional Reallocation Across Variability Groups

**Figure 7:**
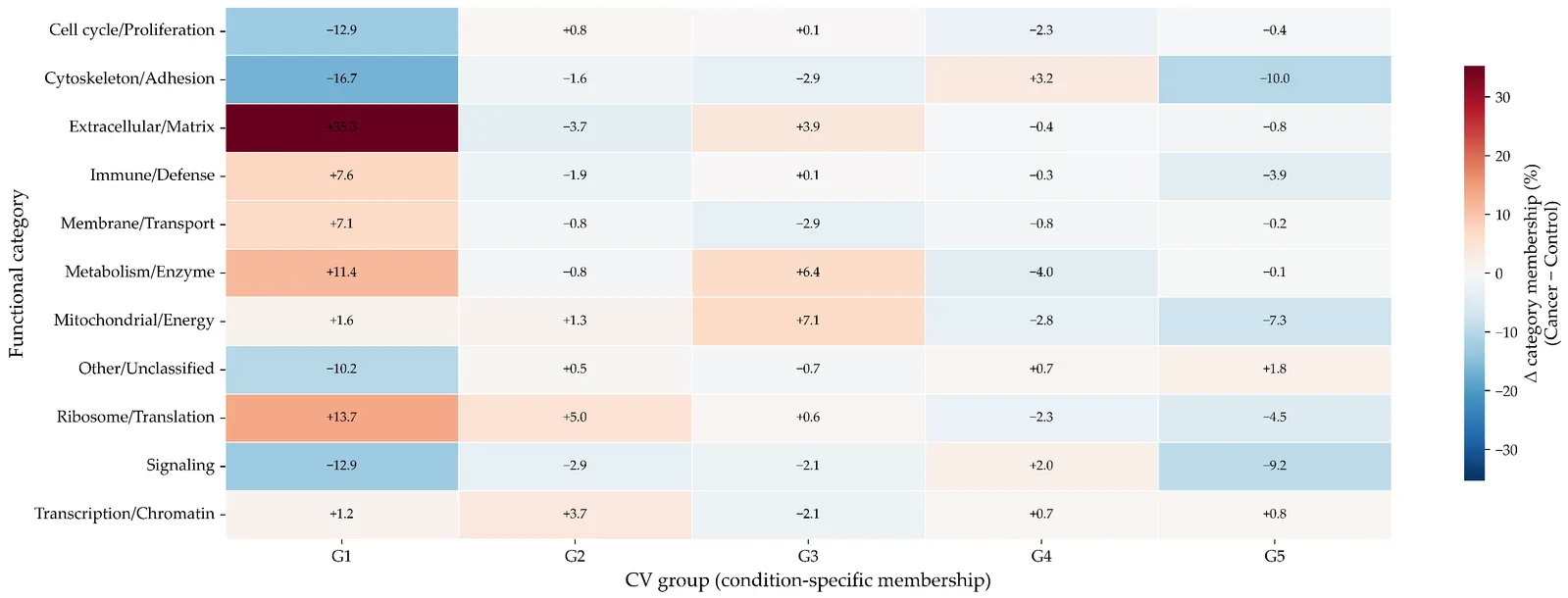
Functional reallocation across variability-defined protein groups (cancer − control). Heatmap showing differences in functional category representation (cancer − control, %) across control-defined CV groups (G1–G5). Rows correspond to functional categories, and columns represent CV groups defined based on control variability. Positive values (red) indicate increased representation in cancer, while negative values (blue) indicate decreased representation.

Functional composition analysis revealed non-uniform changes across variability-defined groups (Figure 7). The strongest shifts were observed in the lowest-variability proteins (G1), which showed increased representation of extracellular and metabolic categories and decreased representation of structural and signaling-related functions in the cancer group. In contrast, proteins with higher baseline variability (G4–G5) exhibited smaller and more heterogeneous changes. These results indicate that functional reorganization is concentrated in proteins that are stable in the control group, rather than uniformly distributed across the proteome.

The most pronounced shifts are observed in the lowest-variability group (G1), where the cancer group is associated with a strong increase in extracellular/matrix proteins (+35.3 percentage points) and concurrent decreases in cytoskeleton/adhesion (−16.7), cell cycle/proliferation (−12.9), and signaling (−12.9). Proteins in G1 show a pronounced increase in CV between control and cancer (Section 2.2). Project-adjusted limma was applied independently to the ≥50% detection universe (*n* = 4695; Supplementary Figure S1 and Supplementary Table S1). At BH FDR < 0.05, 674 proteins were differentially abundant (550 higher and 124 lower in cancer; median log2 fold change = 0.57). CV groups were assigned only after limma significance testing; 327 of the 674 limma-positive proteins belonged to either of the two pre-specified CV groups and are marked by green rings. Thus, CV grouping was not used to filter the abundance test. This partial overlap indicates that abundance and variability provide complementary descriptions, but it is not independent validation because both analyses use the same samples. Intermediate groups (G2–G3) show more moderate and category-specific changes than G1. Metabolic and energy-related functions increased further in G3 (metabolism/enzyme: +6.4; mitochondrial/energy: +7.1), while G2 showed comparatively little change across most categories (|Δ| ≤ 5 percentage points for all but ribosome/translation, +5.0), including a slight decrease in metabolism/enzyme (−0.8). In higher-variability groups (G4–G5), changes are smaller and less coordinated, with a general reduction in several categories at the highest variability level (e.g., cytoskeleton/adhesion: −10.0; signaling: −9.2; mitochondrial/energy: −7.3 in G5).

### 2.8. Limitations

Several limitations of this study should be considered when interpreting the findings. First, the reliability of coefficient-of-variation estimates depends on the number of available observations. Although a minimum threshold of three non-zero values per group was applied, CV estimates for low-intensity and rarely detected proteins may remain unstable and sensitive to individual measurements. Zero values were treated as missing and excluded from the calculations, which reduces the influence of non-detected signals on the mean but may bias variability estimates for proteins with a high proportion of missing values. In addition, this approach excludes proteins that are not detected in the control group but are present in cancer samples, although such proteins may represent potentially informative disease-associated markers. This limitation reflects a recognized challenge in high-throughput omics data preprocessing, as the strategy used to handle missing and undetectable values can substantially affect subsequent statistical analyses [13].

Second, normalization to the total protein signal assumes that the overall proteomic composition remains reasonably comparable across samples. Changes in a small number of highly abundant proteins may create compositional effects and influence the normalized values of the remaining proteins. Although this approach reduces differences in total protein loading and overall MS signal intensity, it may also affect estimates of within-group dispersion. The robustness of the observed patterns should therefore be further evaluated using alternative normalization methods, including median scaling and variance-stabilization approaches [9,28].

The heterogeneity of the integrated public datasets should be taken into account. The included projects differed in biomaterial type, tissue preservation, sample preparation, analytical platform, cohort size, clinical composition, and depth of proteomic coverage. To harmonize protein identification and quantification, RAW files from all four projects were reprocessed using the same MaxQuant v2.4.7.0 workflow with the Andromeda (v2.4.7.0, integrated within MaxQuant) search engine against UniProtKB Human (release 2025_01). Nevertheless, uniform computational processing cannot eliminate specimen-, instrument-, project-, and cohort-specific effects. Mass-spectrometric protein intensities also depend on peptide-specific ionization efficiency, chromatographic behavior, charge state, ion transmission, matrix suppression, and proximity to the detection limit [29,30]. Consequently, measured CV reflects a combination of inter-individual biological heterogeneity and residual pre-analytical and technical variability, particularly for low-abundance proteins.

Increased protein variability is also not specific to malignancy and may accompany inflammation, immune activation, fibrosis, aging, and other pathological or physiological conditions [31]. The present findings therefore indicate cancer-associated reorganization of proteomic variability but do not, by themselves, establish the diagnostic specificity or predictive performance of individual proteins or CV-based signatures.

Age represents an additional potential confounding factor because ovarian carcinoma predominantly occurs in postmenopausal women [32]. Recent evidence indicates that the abundance and variability of individual proteins may differ systematically across age groups even in healthy populations [33]. Age-related shifts in baseline variability should therefore be considered when interpreting cancer-associated changes in CV. Furthermore, the current analysis treated the patient cohorts as relatively homogeneous because information on menopausal status, tumor stage, grade, molecular subtype, treatment, therapeutic response, and survival was incomplete or unavailable for some public datasets.

Future studies should therefore include age- and menopause-matched cohorts and apply stratified analyses according to clinically relevant characteristics. Extending the CV-based framework to subgroups such as chemotherapy responders and non-responders, patients with different molecular subtypes, or patients with distinct clinical outcomes may reveal variability signatures with prognostic or therapeutic relevance. Independent validation in well-characterized cohorts, including benign and inflammatory control conditions, together with direct assessment of sensitivity, specificity, and classification performance, will be required before these features can be considered for precision diagnostic or therapeutic applications. Project-specific descriptive comparisons and leave-one-project-out sensitivity analyses were performed (Supplementary Figures S4–S6; Supplementary Tables S3 and S4). The leave-one-project-out results showed marked project heterogeneity and are treated as sensitivity analyses rather than independent validation. A formal project-level meta-analysis was not performed.

## 3. Discussion

Using Protein Variability Pattern analysis as a distinct proteomic feature framework, we show that ovarian cancer is characterized not by a uniform increase in protein-level variability but by its redistribution across proteins and functional categories. Increased inter-individual variability is not unique to ovarian cancer and is expected in malignant tissues with substantial cellular and microenvironmental heterogeneity. The relevant question is therefore not whether cancer is associated with greater variability, but whether the pattern of variability contains structured information beyond changes in mean protein abundance. In our analysis, proteins differed in both the magnitude and direction of CV changes, with low-variability proteins tending to become more heterogeneous and highly variable proteins showing more heterogeneous trajectories, including partial stabilization.

This interpretation is consistent with the emerging view that loss of homeostatic control in malignancy may manifest as altered molecular dispersion, a phenomenon recently systematized at the transcriptomic level through resources such as The Cancer Noise Atlas (TCNA) [34]. If changes in regulatory control are reflected in molecular dispersion, a similar principle may also operate at the protein level. Clinical laboratory studies provide an additional context for interpreting such variability. For example, serum anti-Müllerian hormone in healthy adult men showed a within-subject CV of 3.5% but a between-subject CV of 34.72%, illustrating that tightly regulated individual homeostatic levels may nevertheless differ substantially across a population [35]. Similarly, red cell distribution width (RDW), mathematically defined as the CV of erythrocyte volume, is tightly regulated within individuals but becomes elevated in systemic inflammation and various malignancies and can serve as an independent prognostic marker [36]. These observations support the use of CV as a biologically informative property, while also emphasizing that increased variability itself is not specific to ovarian cancer.

This idea is supported by the natural variability of protein expression between cells. Protein levels are not completely constant and may differ even among genetically similar cells. As demonstrated by Sigal et al. [37], the levels of 20 endogenous proteins in living human cells varied by 15–30% around the mean, with transitions between high and low levels occurring slowly and often spanning more than two cell generations (>40 h). Proteins belonging to the same biological pathway also exhibited more coordinated levels, suggesting that the long-term “memory” of protein abundance may influence individual cellular behavior and the timescale of population responses to external signals. The present study extends this concept from variability among individual cells to inter-individual variability across patient proteomes. Our results show that, in ovarian cancer, variability is redistributed across proteins and functional groups. Proteins with low variability in the control group tend to become more variable in the cancer group, whereas highly variable proteins frequently show partial stabilization. This bidirectional behavior is supported by both distributional analysis and transition matrices, indicating that cancer-associated variability reflects structured reorganization rather than simple variance inflation. In particular, Arora et al. [38] showed that metastatic progression of ovarian cancer is accompanied by a systemic reorganization of metabolic processes at the proteome level, which indicates the functional orientation of the observed changes.

At the protein level, this effect is heterogeneous [39]. Even within the same baseline variability class, proteins exhibit different trajectories, including increases, decreases, and relatively limited changes in CV. The present results are consistent with the spatial proteomic analysis of breast cancer conducted by Mardamshina et al. [40]. The authors showed that the heterogeneity of proteomic profiles cannot be fully explained by genetic differences and also reflects cellular functional states and characteristics of the tumor microenvironment. This suggests that variability changes are not merely group-driven artifacts but may reflect protein-specific differences in the magnitude and direction of variability changes.

Despite this heterogeneity at the individual-protein level, the observed changes were not functionally random. Functional analyses revealed a clear organization of variability across the proteome. Low-variability proteins were enriched in intracellular and structurally constrained compartments, including the cytoplasm and organelle-associated systems. In contrast, high-variability proteins were more frequently associated with membrane, vesicle, and extracellular processes. This pattern is consistent with the concept of conserved modules described by Ergin et al. [41], according to which a core set of proteins supporting fundamental cellular functions maintains stable expression levels across different types of tumor cells. These functional distinctions are also consistent with known biological differences between core cellular machinery and interface-driven systems that are more responsive to environmental and pathological variation.

Proteins that changed their variability also differed in function. Proteins that became more variable in the cancer group were often involved in regulation, response to stimuli, and vesicle-related processes. In contrast, proteins that became less variable were more often associated with intracellular functions.

Together, these results show that cancer changes how protein variability is distributed across the proteome. Intracellular proteins generally remain more stable, whereas membrane-associated and extracellular proteins show greater variability. This pattern may reflect differences in the tumor microenvironment, immune activity, and signaling processes.

Variability analysis therefore provides information that is complementary to standard differential-abundance analysis. Differential-abundance analysis measures changes in mean protein levels, whereas CV analysis shows how strongly protein levels differ between individuals. A protein may have a similar mean level in control and cancer but show a marked increase in variability. Such changes may help identify proteins associated with tumor heterogeneity that would not be detected by mean-based comparisons alone.

The analysis also identified a relatively stable proteomic “core” and a more variable group of proteins. Stable proteins may serve as internal reference proteins, whereas proteins that become more variable in the cancer group may represent candidates for further investigation. However, increased variability alone is not sufficient to define a clinically relevant biomarker.

CV must also be interpreted in relation to the mean signal level. Low-intensity proteins often show high relative variability because of limited detection and technical noise. In transcriptomics, this problem is addressed by modeling the relationship between the mean and variance or by applying variance-stabilization methods [16,17,42,43]. For proteomic data, this implies that a high CV for a low-intensity protein does not necessarily reflect biological instability and may partly be determined by the proximity of the signal to the detection limit.

Protein coverage represents an additional factor that may affect the reliability of CV estimates. In mass-spectrometry proteomics, proteins quantified using a limited number of peptides or PSMs may yield less stable estimates of abundance variability, whereas greater quantitative coverage generally provides more reliable variance estimates [44]. This issue should be considered when interpreting the present results, as the analyzed datasets differed in proteomic depth and analytical platform. Therefore, part of the observed differences in protein-level CV may reflect differences in quantitative coverage in addition to biological heterogeneity.

An additional source of variability is the normalization approach [9,45,46]. The total-protein-signal scaling used in this study reduces differences in overall sample intensity but does not fully eliminate compositional effects. Changes in a few high-intensity proteins can affect the relative values of many other proteins. Systematic studies of label-free proteomics have shown that the choice of normalization method can substantially alter within-group dispersion and the results of subsequent statistical analysis [47,48].

In mass spectrometry, protein quantification is based on the signals of proteolytic peptides. Peptide intensity is determined not only by its abundance but also by electrospray ionization efficiency, charge state, chromatographic elution, ion transmission, and matrix suppression [29,30,49]. Consequently, equal molar amounts of different peptides can produce substantially different MS signals. Moreover, differences in the composition of tumor and control samples may alter the degree of ion suppression for the same peptide.

Thus, the observed CV reflects a combination of biological heterogeneity and residual technical variability. Biological interpretation of CV changes is most justified when the identified patterns persist after adjusting for mean intensity, detection frequency, normalization strategy, and project-specific technical factors. Protein variability pattern-focused analysis should therefore be regarded as a complementary aspect of proteomic data analysis, rather than a replacement for standard mean-abundance and fold-change analysis.

Prior to analyzing potential biomarkers, the detectability and variability of each protein must be assessed [50,51,52]. Low ionization efficiency, ion suppression, and limited sensitivity of mass spectrometry can lead to inconsistent peptide detection and missing values even when the corresponding protein is present in the sample. Furthermore, high analytical or interindividual biological variability increases the overlap of distributions between clinical groups and reduces the statistical power to detect disease-associated changes. In contrast, proteins that are consistently detected and characterized by low intra-group variability provide a more reproducible quantitative signal and represent more reliable candidates for subsequent diagnostic analysis.

## 4. Materials and Methods

### 4.1. Study Design

Protein-level coefficient of variation (CV) was quantified across Control and Cancer samples from four ovarian cancer proteomic datasets. The analysis comprised CV estimation and assignment to predefined variability groups, followed by functional annotation and enrichment analysis. The reproducible workflow was implemented in Python 3.12.10 and R 4.6.1.

### 4.2. Datasets

Four publicly available LC-MS/MS label-free proteomic projects were analyzed (Table 1): PXD009655 [21], PXD012998 [22], PXD025864 [23], and PXD033741 [24]. RAW mass-spectrometry files and control/cancer labels were obtained from the project records. The RAW files were reprocessed using a common MaxQuant workflow before the resulting protein-group/iBAQ matrices were integrated for downstream analysis. Clinical and histological metadata were incomplete and could not be harmonized uniformly across projects; Table 1 therefore reports only consistently available fields, and the missing metadata are treated as a limitation.

These projects represent distinct experimental designs, specimen types, preservation procedures, cohort sizes, proteomic coverage, and mass-spectrometry platforms (Table 1). RAW files from each project were processed separately by project using the same MaxQuant v2.4.7.0 configuration and protein database, after which a common downstream workflow was applied to the resulting protein-group/iBAQ matrices. Thus, search-engine, database, FDR, filtering, and quantification settings were harmonized, while instrument-, specimen-, and project-specific effects remained. FFPE tissue, liquid biopsy, fresh-frozen tissue, Orbitrap instruments, and timsTOF Pro may contribute different technical and pre-analytical variance. Measured CV therefore combines biological heterogeneity with residual pre-analytical and technical variability, and project-aware sensitivity analyses were used to assess, but cannot eliminate, this limitation.

Only samples labeled as control or cancer were retained. The final dataset included 44 control samples and 136 cancer samples, corresponding to 180 biological sample-level profiles after technical replicate aggregation. Unequal sample sizes also reflect the structure of available clinical data on high-grade serous carcinoma (HGSC).

Although estimating dispersion metrics is statistically more demanding than estimating mean abundance, the control cohort size (*n* = 44) provides a stable baseline for CV estimation. Adequate biological replication is essential for reliable estimation of biological variance [53]. Uncertainty in cohort-level CV summaries was assessed using 10,000 sample-level bootstrap replicates with resampling within each project × condition stratum. The bootstrap median was 83.7% in control (95% CI 77.0–89.0%) and 116.6% in cancer (95% CI 112.1–121.1%); the bootstrap estimate of the difference between median cancer and control CV was 33.0 percentage points (95% CI, 25.9–41.0 percentage points). These intervals quantify sampling uncertainty in cohort-level summaries and are not protein-specific confidence intervals (Supplementary Figure S7 and Supplementary Table S5).

Large projects, such as PXD033741, significantly exceed independent external cohorts in terms of sample numbers. In contrast, smaller studies may involve a limited number of patients but often include valuable normal controls or specific types of biomaterial. An additional challenge is the scarcity of normal fallopian tube tissues, which results in the number of control samples being considerably lower than tumor samples in nearly all open epithelial ovarian cancer projects.

### 4.3. Quantitative Matrix Acquisition and Upstream Processing

RAW mass-spectrometry files from PXD009655, PXD012998, PXD025864, and PXD033741 were downloaded from the public project records and processed separately by project using the same MaxQuant v2.4.7.0 workflow with the Andromeda search engine against UniProtKB Human (release 2025_01). Peptide- and protein-level FDR were controlled at <1%; reverse hits, potential contaminants, and proteins identified only by site were removed; iBAQ values were used for label-free quantification. Protein inference, shared-peptide handling, decoy filtering, and isoform grouping followed MaxQuant protein-group rules under the common configuration. The resulting protein-group matrices were merged, and the downstream workflow comprised technical-replicate aggregation, sample-wise normalization, detection filtering, identifier mapping, CV calculation, and statistical analysis. No missing-value imputation was applied. DEqMS was not used because harmonized peptide/PSM-count covariates were not retained in the final merged analysis matrix.

### 4.4. Protein Intensity Matrix

The input matrix contained protein groups as rows and samples as columns, with entries representing normalized protein intensities.

All analyses were conducted on non-log-transformed data. Log transformation compresses variance, particularly at higher abundance levels, and may obscure dispersion patterns when variability is the primary quantity of interest. Therefore, CV was computed on the original normalized scale to preserve the relationship between mean and variance.

### 4.5. Technical Replicate Aggregation

Some biological samples were measured multiple times for quality control. Technical replicates were identified by matching sample identifier, disease status, project, and tissue type.

For samples with multiple measurements (*n* > 1), protein intensities were aggregated using the median. This approach reduces sensitivity to outliers and ensures robust estimation across heterogeneous datasets. Samples with a single measurement were retained without modification.

### 4.6. Sample-Wise Normalization

Protein intensities were normalized within each sample to account for differences in total protein loading and overall MS signal intensity:

I_norm_ = I_protein_/Σ I_sample_

where I_protein_ is the intensity of a given protein and Σ I_sample_ is the total intensity across all proteins in the sample. The normalized value represents each protein’s relative contribution to the total MS signal in that sample.

This normalization transforms absolute intensities into relative proportions, reducing global technical variation while preserving biological differences in protein composition. Normalization was applied prior to downstream variability analysis.

The resulting value reflects the relative contribution of a given protein’s signal to the total detected proteomic signal of the sample and should not be interpreted as the absolute intracellular protein concentration. Normalization was performed prior to calculating coefficients of variation.

Normalization to the total signal reduces global differences between LC–MS/MS runs but may be sensitive to compositional effects. If a small number of high-intensity proteins account for a substantial share of the total signal, changes in their abundance affect the denominator and, consequently, the normalized values of all other proteins. Similar limitations of total-signal normalization have been described in both transcriptomics and quantitative label-free proteomics [9,28,45].

### 4.7. Coefficient of Variation Calculation

CV was calculated separately for the control and cancer groups according to the standard definition:

CV(%) = (σ/μ) × 100,

where σ is the standard deviation calculated using *n* − 1 degrees of freedom, and μ is the mean protein abundance across samples within each condition. Only positive intensity values were used for CV estimation; zero values were treated as missing observations and excluded from the calculation. Proteins were required to have at least three non-zero measurements within each condition. Because CV scales the standard deviation by the mean abundance, it facilitates comparison of dispersion across proteins spanning different abundance ranges. CV is undefined when the mean equals zero and may be unstable under sparse detection. The ≥3-positive criterion was retained for the primary analysis, and detection-proportion thresholds of 50%, 70%, and 80% were evaluated as sensitivity analyses. Proteins detected predominantly in one condition were tabulated separately and were not interpreted as paired CV transitions.

For each protein, condition-specific coefficients of variation (CV_Control_ and CV_Cancer_) were calculated together with the signed difference in variability (ΔCV = CV_Cancer_ − CV_Control_). In addition, proteins were assigned to predefined variability categories in both conditions, allowing subsequent analysis of transitions between variability states. After filtering, 7545 proteins had valid CV estimates in the control group, and 7476 proteins had valid estimates in both control and cancer; the latter set was used for all comparative analyses.

### 4.8. CV Group Classification

Proteins were classified into five variability groups based on control CV values (Table 2).

These fixed thresholds provide a consistent framework for comparing variability levels across proteins. The same thresholds were applied to cancer CV values to assess transitions between variability states.

Proteins were classified according to relative changes in variability using the ratio CV_Cancer_/CV_Control_. Three categories were defined: increased variability, ratio > 1.5; approximately unchanged variability, 0.67 ≤ ratio ≤ 1.5; and decreased variability, ratio < 0.67. These thresholds identify substantial directional changes while minimizing sensitivity to minor fluctuations and measurement noise.

### 4.9. Functional Annotation

Protein identifiers were mapped to functional annotations including gene names, subcellular localization, pathway information, and Gene Ontology (GO) terms. When pathway annotations were incomplete, pathway-related descriptors were extracted from GO annotations.

Proteins were assigned to ten broad functional categories: transcription/chromatin; extracellular/matrix; signaling; metabolism/enzyme; immune/defense; mitochondrial/energy; ribosome/translation; cytoskeleton/adhesion; membrane/transport; and cell cycle/proliferation.

Assignment was performed using keyword-based matching, allowing multi-label annotation and enabling functional profiling across CV-defined groups. The category percentages underlying Figure 5 are provided in Supplementary Table S9, and the reproducible implementation is included in the Zenodo code archive.

### 4.10. Statistical Analysis

Sampling uncertainty for pooled CV summaries and transition proportions was estimated using 10,000 profile-level bootstrap resamples performed within project and condition. Transition inference used 100,000 project-stratified label permutations (seed = 42), with the Stable set redefined in every permutation. The two pre-specified primary endpoints were the conditional proportions of Stable Retained and Destabilized proteins among proteins classified as Stable in pseudo-control; one-sided empirical *p* values were adjusted by the Benjamini–Hochberg procedure across these two endpoints. Absolute counts and individual transition-matrix cells were treated as secondary or descriptive outputs.

Split-half reproducibility was assessed with 10,000 project-stratified resamples using Spearman rank correlation, exact group concordance, weighted kappa, and group-specific Jaccard overlap. To evaluate regression to the mean, empirical-Bayes shrinkage was applied to log-transformed CV estimates for all 7476 proteins before G1–G5 reassignment; raw and shrunk classifications were compared by counts and Jaccard overlap.

For LOPO sensitivity analysis, one project was excluded at a time and the valid protein universe, CV values, and G1–G5 assignments were recomputed. Two primary endpoints were tested using 10,000 project-stratified label permutations: the two-sample Kolmogorov–Smirnov distance for the complete CV distribution and total-variation distance for the marginal G1–G5 composition. Benjamini–Hochberg correction was applied across the eight primary tests (two endpoints × four exclusions). Stable → Stable and Stable → Variable endpoints formed a separate secondary family of eight tests. LOPO results are reported in Supplementary Figure S6 and Supplementary Table S4.

Protein-level differential-variability screening was performed for the pre-specified family of all 4024 proteins classified as Stable in control. For each protein, signed ΔCV and log2(CV_Cancer_/CV_Control_) were reported as effect measures. Empirical *p* values were estimated from 10,000 project-stratified label permutations, with a protein contributing only when it was reclassified as Stable in pseudo-control. Benjamini–Hochberg correction was applied across the complete 4024-protein family; an exploratory candidate flag additionally required at least 1000 accepted conditional permutations. Protein-level results are reported in Supplementary Table S6 and are treated as hypothesis-generating rather than independent validation.

#### 4.10.1. Differential-Abundance Analysis

Differential abundance was assessed with project-adjusted limma on proteins detected in at least 50% of samples in both conditions (*n* = 4695). The model was log2 intensity ~ status + project, with empirical-Bayes moderation and Benjamini–Hochberg correction. Significance was defined as FDR < 0.05. Limma was run before any CV-group annotation; the two pre-specified CV groups were overlaid only after significance testing.

#### 4.10.2. Enrichment Analysis

Gene Ontology enrichment analysis was performed using g:Profiler [54] with three ontology sources: GO Biological Process (GO:BP), GO Molecular Function (GO:MF), and GO Cellular Component (GO:CC). Four protein sets, defined by control CV group and by control-to-cancer transition, were analyzed: stable proteins (G1–G2, *n* = 4024 proteins; 3978 mapped gene symbols used as the g:Profiler query); variable proteins (G4–G5, *n* = 1590 proteins; 1578 gene symbols); destabilized proteins (control-stable to cancer-variable, *n* = 733 proteins); and stabilized proteins (control-variable to cancer-stable, *n* = 219 proteins; 216 gene symbols). Enrichment for each GO term was assessed using g:Profiler’s built-in hypergeometric test against a custom background comprising the 7476 proteins with valid CV in both control and cancer conditions (7470 mapped gene symbols; g:Profiler effective domain size = 7522). Multiple testing correction was performed using the Benjamini–Hochberg procedure to control the false discovery rate (FDR). Results were filtered at FDR < 0.05.

### 4.11. Visualization

All visualizations were generated in Python 3.12.10 using the Matplotlib 3.11.1 and exported at a resolution of 900 dpi.

## 5. Conclusions

This study showed that inter-sample protein variability in HGSC changes according to baseline CV and is accompanied by differences in functional-category representation. The analysis included 7476 proteins quantified in 44 control and 136 cancer samples. The CV distributionin the cancer group shifted toward higher values. The most pronounced changes occurred among initially stable proteins: in G1, only 4.1% remained within the original CV range, whereas 42.9%, 18.4%, 24.5%, and 10.2% shifted to G2, G3, G4, and G5, respectively. Conversely, 66.7% of proteins in G5 shifted to groups with lower CVs.

Protein groups with different levels of variability also differed in functional-category representation. The proportion of membrane/transport proteins increased from 42.9% in G1 to 65.7% in G5, whereas the proportion of transcription/chromatin proteins decreased from 38.8% to 21.0%. Stable proteins (G1–G2) were enriched mainly in intracellular and organelle-associated terms, whereas variable proteins (G4–G5) showed greater enrichment of vesicular and extracellular terms. No GO term passed FDR < 0.05 for proteins shifting from G1–G2 to G4–G5, while the reverse transition yielded two significant terms: nucleic acid binding (FDR = 2.93 × 10^−4^) and rRNA metabolic process (FDR = 0.0322).

Thus, CV analysis complements comparisons of mean protein abundance by accounting for inter-sample variability when evaluating differences between control and cancer groups. At the class level, neither pre-specified transition proportion exceeded the project-stratified null after FDR correction (Stable Retained, FDR = 0.422; Destabilized, FDR = 0.071), although the Destabilized class was stable under CV shrinkage. Ninety-five individual proteins met the exploratory family-wide differential-variability criterion, but they require independent validation. LOPO analysis demonstrated project heterogeneity rather than uniform replication. Independently, project-adjusted limma identified 674 differentially abundant proteins at FDR < 0.05. These findings support variability and abundance as complementary, hypothesis-generating descriptions of the HGSC proteome.

## Figures and Tables

**Table 1:** Summary of the four label-free LC-MS/MS proteomic datasets included in this study. Detailed dataset descriptions are provided in Supplementary Table S11.

Project ID	Number of Samples (HGSC/Control)	Sample Type	Material Source	Mass Spectrometry Platform
PXD009655	50 (43/7)	Liquid biopsy	Utero-tubal lavage	Q-Exactive Plus or Q-Exactive HF
PXD012998	14 (10/4)	Tissue	Fresh frozen ovarian and fallopian tube tissue	Orbitrap Elite or Q-Exactive Plus
PXD025864	6 (3/3)	Tissue	Fresh frozen fallopian tube tissues	Q-Exactive
PXD033741	110 (80/30)	Tissue	FFPE * ovarian tumor and control (uterine fibroids) tissue	timsTOF Pro

* Formalin-fixed paraffin-embedded (FFPE) tissue.

**Table 2:** Classification of proteins into variability groups based on coefficient of variation thresholds in control samples.

Group	CV Range	Description
G1	0–30%	Very low variability
G2	30–100%	Low variability
G3	100–200%	Intermediate variability
G4	200–400%	High variability
G5	>400%	Very high variability

## Data Availability

The data presented in this study are available in the article, its Supplementary Materials, and the associated Zenodo record (https://doi.org/10.5281/zenodo.21307702). The archive contains the Graphical Abstract, main Figure 1, Figure 2, Figure 3, Figure 4, Figure 5, Figure 6 and Figure 7, Supplementary Figures S1–S10, Supplementary Tables S1–S11, input data, complete Python and R analysis code, and README files.

## Supplementary Materials

The following supporting information can be downloaded at: https://www.mdpi.com/article/10.3390/ijms27188012/s1.
